# The Effect of Social Networks on Active Living in Adolescents: Qualitative Focus Group Study

**DOI:** 10.2196/46350

**Published:** 2023-10-05

**Authors:** Sander Hermsen, Femke Van Abswoude, Bert Steenbergen

**Affiliations:** 1 Precision Health and Nutrition Group OnePlanet Research Centre Wageningen Netherlands; 2 Behavioural Science Institute Radboud University Nijmegen Nijmegen Netherlands

**Keywords:** active living, adolescents, physical activity, digital health, mobile phone

## Abstract

**Background:**

Participation in organized sports and other forms of active living have important health benefits in adolescence and adulthood. Unfortunately, the transition to secondary school has been shown to be a barrier to participation. Social networks can play important roles in activating adolescents, and information and communication technology (ICT) interventions can augment this role. To date, there are few insights into what adolescents themselves think and feel about barriers to and motivators for active living, the role of their social networks in active living, and the potential of ICT for physical activity (PA).

**Objective:**

This study aimed to gather insights into the perspectives of adolescents aged 12 to 14 years on active living and sports participation, motivators and demotivators for active living, and the potential roles of their social network and of ICT.

**Methods:**

A total of 26 adolescents aged 12 to 14 years from different levels of Dutch secondary schools participated in 1 of 5 semistructured focus group interviews, in which they talked about sports and PA, their social networks, their ICT use, and the role of social networks and ICT in PA. All interviews were transcribed and analyzed using a thematic qualitative approach.

**Results:**

The study showed that all participants were physically active, although the transition to secondary school made this difficult, mostly because of time constraints. Participants saw positive physical and mental health effects as important benefits of active living. They regarded social benefits as strong motivators for active living: being together, making friends, and having fun together. However, the social network could also demotivate through negative peer judgment and negative feedback. Participants were willing to share their own positive experiences and hear about those from close peers and friends but would not share their own (and were not interested in others’) negative experiences or personal information. Participants were mainly interested in descriptive norms set by others and obtained inspiration from others for PA. With respect to using ICT for active living, participants stated a preference for social challenges among friends, personalized feedback, goals, activities, and rewards. Competition was seen as less important or even unattractive. If mentioned, participants felt that this should be with friends, or peers of a similar level, with fun being more important than the competition itself.

**Conclusions:**

This study shows that adolescents feel that their social network is and can be a strong driver of active living. They are willing to use ICT-based solutions that make use of social networks for PA as long as these solutions involve their current (close) network and use an approach based on being together and having fun together.

## Introduction

### Background

Physical inactivity is one of the most important threats to individual health worldwide and is the number one factor for all-cause mortality [1-5], contributing to >5 million premature deaths each year [1]. An inactive lifestyle is caused by both a lack of involvement in sports activities and other leisure-time physical activities (PAs) and sedentary behavior in the contexts of work, school, and home. The benefits of sufficient PA (see recent World Health Organization guidelines [5]) for health, such as cardiometabolic health [6], overall physical fitness (eg, strengthening bones [7]), and mental well-being [8,9] are well known; however, PA also benefits people in a broader range of other aspects of life (“human capitals” [10]). Adequate PA benefits people individually (eg, by developing goal setting and project management skills [11]), intellectually (eg, cognitive capacity and improved concentration [12]), financially [13], and socially (eg, development of social skills [9]). A lack of PA, by contrast, is strongly associated with a range of debilitating conditions such as type 2 diabetes, cardiovascular disease, and mental health problems [1,4].

The benefits of PA are especially pronounced in children and adolescents, because adequate PA not only affects their immediate health status, such as positive changes in adiposity, skeletal health, psychological health, and cardiorespiratory fitness [14,15], but also has a great impact on their future health. An improved cardiovascular profile provides continued benefits in adulthood [14]. In addition, motor skill development during early childhood has long-lasting effects in adulthood, and the benefits of PA during childhood also positively influence adult health outcomes, such as increased bone mineral density [15]. In addition, PA and fitness during adolescence significantly predict PA in adulthood, making it an important contributing factor to a lifelong active lifestyle [16].

Given the strong evidence for the benefits of PA, and the role it could play in preventing debilitating health conditions and the promotion of healthy lifestyles, it is vital to understand the facilitators of and barriers to an active lifestyle in childhood and adolescence [17]. This understanding can help build interventions to support people in starting and maintaining sports participation, exercise, or active daily habits such as bicycle commuting, walking, or performing regular PA in the workplace. Fortunately, a broad body of literature identifies the determinants of adolescents’ PA, such as sex (males more likely to do PA than females), ethnicity (White individuals more likely to engage in PA than other groups), age (the older the individual, the lesser the PA), socioeconomic status (people with higher status have higher PA levels), perceived self-efficacy, previous PA, availability of community sports facilities and other opportunities to exercise, sedentary behavior after school or on weekends, parental support, support from others, PA of siblings, having paid work (less PA), and media use [18-20]. Age is one of the most important determinants of a child’s PA, with adolescents consistently being less active than younger children [21-23].

One specific, well-researched factor that affects PA is the impact of life transitions. Generally speaking, life transitions are all known moments in which people are most at risk of adopting a less physically active lifestyle [24-26]. Several such life transitions exist, such as moving from primary school to secondary school, moving out of the parental house, starting a working career, becoming a parent, and retiring from working life. Other factors leading to a less-active lifestyle related to life transitions are time and financial constraints, other (competing) interests, (perceived) lack of energy, and negative social influences [27-29]. Adolescents in most countries around the world experience one of these life transitions, that is, when they enter secondary school, usually between their 10th and 16th birthdays; evidence [30-33] shows that this life transition, entering secondary school, indeed contributes to reduced PA.

To support adolescents to be active, a broad range of interventions exist [34,35]. An interesting and as yet underresearched approach for the development of these interventions is to use social networks. Social networks act as strong facilitators of PA [36-40]. This effect is especially visible in adolescents, whose PA level is directly and significantly associated with that of their friends [15,41-43]. This dependency is reciprocal: PA leads to friendships, which in turn affect PA [41]. Social networks can support individuals by providing (descriptive or injunctive) positive social norms regarding PA [44-46], by offering possibilities for vicarious learning [47,48], by offering (practical and emotional) social support [45,46,49,50], by providing feedback on an individual’s PA status and performance [51,52], and by offering shared experiences [53].

Information and communication technologies (ICTs) offer previously unseen opportunities to tap into adolescents’ social networks. In recent years, 90% to 95% of teens in Western countries have reported having access to and regularly using a smartphone [54,55], with no differences between sex, ethnicity, and socioeconomic backgrounds. Smartphones play an essential role in connecting the individual with their social networks, and these technologies therefore also impact the PA of the individual and the group [56-61]. In recent years, ICT-based solutions have been used for mapping social systems [62,63] and for sensing the aspects of individual, pair, and group behavior in networks, such as colocation, communication and interaction, and coacting [64]. Furthermore, ICTs have been used to enable the targeting of interventions aimed at certain members of the network [65] and to enhance social networks, thereby fostering behavior change [50]. When used for behavior change interventions, ICTs in the form of apps, websites, and wearables can be useful in delivering interventions to help people identify and adapt to descriptive and injunctive social norms [46,66,67]: seeing others’ PA through technological channels can both increase [56] and decrease [43] a person’s own activity because people tend to adjust their performance to perceived descriptive norms. Information about others’ PA also offers opportunities for vicarious learning [68], for instance, by offering context to the data that shape the comparison with others in the norm-setting process. Furthermore, ICTs enable members of a social network to offer feedback and social support to other members [67]. Finally, ICTs enable shared experiences even when people are not physically together at the same location [67,69]. Even without the use or delivery of specific behavior change techniques (BCTs), engagement in the social aspects of ICTs can affect PA behavior. Sharing one’s own data is known to promote self-reflection [70], and wearing or using apps and wearables can augment existing social signaling and self-presentation, thereby augmenting existing behavior [69]. Furthermore, providing social support to other social network members has known health benefits in itself [71]. Recent research has also shed light on what works in engaging adolescents, with rewards; social interactions; varied challenges with tailored difficulty levels; automated self-monitoring; and a variety of customization options (self-set goals, personalized feedback, progress indicators, and customized narrative) as drivers of engagement. Conversely, competition, overly complex instructions, badly timed messages and notifications, technical bugs, and burdensome manual input of self-monitoring act as the barriers to engagement [72].

### Objective

Given the available theory and evidence supporting the importance of active living for adolescents and the potential for (ICT-based) social network interventions to support them in obtaining and sustaining an active lifestyle, it is surprising to find that there is little attention in the current literature regarding what adolescents themselves think and feel about the barriers to and facilitators of active living, the role of their social network, and the potential for ICT-based interventions therein. Therefore, this study aimed to address this gap in the literature. This was done by presenting the results of a qualitative focus group study, in which we asked Dutch adolescents aged between 12 and 14 years who had just entered secondary school, about (changes in) their PA, the role of their peer groups and other social networks in PA, sharing information about active living with their peer groups and others, social media use regarding PA, and their wishes and preferences regarding potential ICT-based interventions.

## Methods

### Overview

This study used a qualitative design using focus group interviews. Focus groups provide a way to collect qualitative data by engaging a small number of people in an informal group discussion, characterized by a focus on prechosen subjects and an interaction between participants [73]. They provide efficient [74], interactive, entertaining [75], and relatively unthreatening [76] settings in which participants can freely discuss their perceptions, ideas, opinions, and thoughts. We had 5 focus groups, each of which consisted of 4 to 7 adolescents, all aged between 12 and 14 years, and in their first or second year of secondary education in the Netherlands. The focus group interviews were held in January and February 2022, a period in which there were relatively few COVID-19–related restrictions. All members of each focus group were in the same class and had the same educational level.

### Recruitment

A total of 14 secondary schools in the Dutch provinces of Overijssel and Gelderland were approached; 2 schools agreed to participate (the reasons given by other schools for not participating were too many requests, time limitations, and pressure on their educational programs due to the COVID-19 pandemic). In each school, 2 classes were selected. In 1 school, the head teacher informed the pupils of the opportunity to participate, stating that the focus group interview would be on PA and how others influence PA. If interested, they could sign up and receive information material and consent forms via email. In the other school, research assistants visited the classes and introduced the research. Pupils were given the information material and informed of the theme of the focus group, and they received a link to the consent forms. Before taking part, all participants and their parents had read the information, had the opportunity to ask further questions, and had given their full consent.

### Procedure

All focus group sessions took place at the pupils’ schools in one of their classrooms. All data were gathered by 4 research assistants as partial fulfillment of their master’s thesis. Each focus group session was led by 3 research assistants, with 1 assuming the role of discussion leader or moderator, whereas the other 2 acted as observers. All the focus group sessions were recorded using a voice recorder. The focus group moderator led the interview by asking 4 main starter questions on sports and PA, web-based and offline social networks and PA, technology and PA, and social media and PA. The moderator asked follow-up questions, if needed. All starter questions and potential follow-up questions are included in Multimedia Appendix 1.

### Sample Size Considerations

A priori sample size calculations in qualitative research are subject to conceptual debates and practical uncertainty. Saturation, that is, the condition when adding more data does not lead to new insights, is often seen as a criterion for the inclusion of more participants once the analysis has started. As a rule of thumb, 20 to 40 participants are usually considered sufficient to achieve saturation in individual interview–based qualitative research [77]. In focus group research, 2 groups are often deemed sufficient for a theme [78], that is, when no new overarching themes emerge; for content or code saturation, that is, when no new information within those themes emerges, more groups are needed. To assess and report thematic and code saturation in this research, we used a method [79] that compared emerging themes and codes within a base set of interviews with those emerging from extra runs of groups. To do so, we used a base size of 3 focus group sessions to allow for theme saturation and then added extra runs of 2 groups until a code saturation level of <5% new information was reached.

### Ethical Considerations

This research was approved by the Ethical Committee of the Faculty of Social Sciences of the Radboud University Nijmegen, The Netherlands (case number ECSW-2021-141), and performed in accordance with the 2013 Declaration of Helsinki.

All interviewees and their parents had ample time to consider their participation, asked questions, and signed informed consent forms. The interview data were stored securely and pseudonymized. The participants received no compensation to participate.

### Reflexivity

Data collection was performed by 4 research assistants, all pedagogical science MSc students, as part of their graduation research. The research questions, interview guide, data collection, and initial thematic analysis rounds were supervised by all the authors. A further round of thematic analysis and refinement of the codebase was performed by all authors.

SH is the principal behavioral scientist at the OnePlanet Research Centre; his work focuses on the acceptability, usability, and efficacy of technological innovations to support people in healthy living. FvA is a postdoctoral researcher and BS is a full-time professor and principal investigator of the active living program at the Radboud University Nijmegen; their work focuses on studying the determinants and opportunities for active living and supporting people in achieving a more active lifestyle.

### Data Analysis

All focus-group recordings were transcribed and checked by the research assistants who led the interview. They anonymized the transcript by removing personal information. All transcripts were then read into the qualitative analysis software, ATLAS.ti (ATLAS.ti Scientific Software Development GmbH) for Windows [80]. These data were then analyzed using an inductive thematic approach and an inductive (open) coding approach. Thematic analysis is a qualitative method used to construct, analyze, and report patterns within textual data [81,82]. This approach is commonly used in qualitative research to identify the key barriers to and facilitators of interventions. The analysis comprised 5 steps. First, all transcripts were split into single units, sentences, or parts thereof containing a single statement. Second, the units from the first transcript received coded classification (“code”). To perform this step, all research assistants first analyzed the transcript individually and applied their own codes, developing their own codebook during the coding process. They then compared their coding to ascertain similar interpretations and developed an updated codebook that integrated all individual codebooks. Third, they coded the remaining transcripts and again compared coding to discuss differences in interpretations and update the codebook. Fourth, to identify emerging patterns and themes, the research assistants grouped similar codes into subcategories and then into themes. Fifth, all transcripts were individually recoded using the finalized coding scheme.

## Results

### Participants

A total of 26 adolescents (females: n=13, 50%; male: n=13, 50%) participated in 5 focus groups of 4 to 7 participants. All participants were aged between 12 and 14 years. The educational backgrounds of the 26 participants differed, with 9 (35%) adolescents attending prevocational secondary education, 5 (19%) participants attending senior general secondary education, and 12 (46%) participants attending preuniversity education. An overview of the descriptive statistics for each focus group session is presented in Table 1.

**Table 1:** Descriptive statistics of the participants attending the focus group sessions (N=26).

Focus group	Participants, n (%)	Participant age range (years)	School level	Sex		School number, region
				Female (n=13)	Male (n=13)	
1	5 (19)	12-13	First year HAVO^a^	1 (8)	4 (31)	1, Twente
2	4 (15)	13-14	Second year VMBO^b^	3 (23)	1 (8)	1, Twente
3	5 (19)	12-13	First year VMBO	2 (15)	3 (23)	1, Twente
4	7 (27)	12-13	First year VWO^c^	4 (31)	3 (23)	2, Gelderland
5	5 (19)	12-13	First year VWO	3 (23)	2 (15)	2, Gelderland

^a^HAVO: senior general secondary education (5 years).

^b^VMBO: prevocational secondary education (4 years).

^c^VWO: preuniversity education (6 years).

All (N=26) participants mentioned participating in sports and other forms of PA (including physical education) at least once a week. Of the 26 participants, only 2 (8%) participants were active only once a week (only the obligatory physical education classes provided by school), and 1 (4%) participant said they were active on every day of the week. Football was by far the most popular sport, with 19 mentions of it. Of the 26 participants, workouts, fitness and gymnastics were mentioned by 4 (15%) participants: free running, golf, scouting, and volleyball by 3 (12%) participants; dancing, cycling, running, hockey, tennis, and swimming by 2 (8%) participants; and athletics, badminton, basketball, boot camp, kickboxing, “klootschieten” (a local ball-throwing game unique to the east of the Netherlands), korfball, motor cross, horse riding, ice skating, and road skating were mentioned once.

### Thematic Analysis

**Figure 1:**
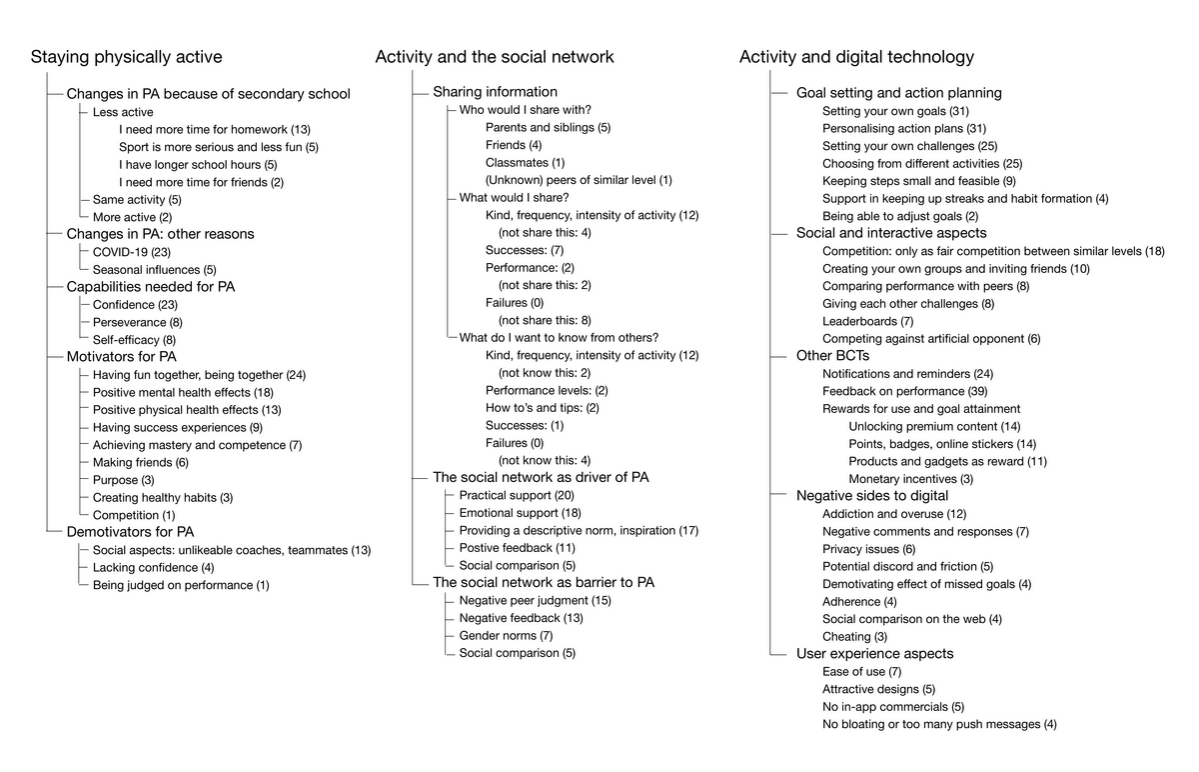
Code overview of all themes constructed and codes derived from the thematic analysis, with code frequency in parentheses. PA: physical activity; BCT: behavior change technique.

Thematic analysis allowed the construction of 3 themes: *Staying physically active*, *PA and the social network*, and *PA and digital technology*. These themes had five, three, and five subthemes, respectively. Staying physically active had the following subthemes: (1) *Changes in PA because of secondary school*, (2) *Changes in PA because of other reasons*, (3) *Perceived capabilities needed for PA*, (4) *Motivators for PA*, and (5) *Demotivators for PA*. The *PA and the social network* theme had the following subthemes: (1) *Who would I share with? What would I share? What do I want to know from others?* (2) *The social network as driver of PA*, and (3) *The social network as barrier to PA*. *PA and digital technology* had the following subthemes: (1) *Goal setting and action planning*, (2) *Social and interactive aspects*, (3) *Other BCTs*, (4) *Negative sides to digital technologies*, and (5) *User experience aspects*. An overview of all themes, subthemes, codes belonging to subthemes, and the frequency of these codes are shown in Figure 1.

### Theme 1: Staying Physically Active

#### Changes in PA Because of Secondary School and Changes in PA Because of Other Reasons

The transition to secondary school brought with it changes in PA for many participants. Many participants explicitly mentioned quitting (one of their) sports activities, and only a few said that they became more active. The increased burden of secondary school was mentioned as the main reason, especially with regard to longer school hours and increased homework (Dutch primary school children have relatively little to no homework and shorter school days); some participants mentioned that they reduce sports activities to be able to spend time with friends:

In primary school, I went out to play every day, after school and in the evening, with friends. And yeah, I cannot do that anymore because of school and homework.

Participant in group 1

I don’t see changes, only that I sometimes have to cancel football or another sport because, well, because school takes up a lot of time.

Participant in group 2

The COVID-19 pandemic has led to an (at least) temporary reduction in PA for all participants due to regulations or quarantines. Interestingly, the COVID-19–related regulations also led to more creative ways of being physically active when organized activities were restricted. Participants also mentioned resuming their activities as soon as possible. Another non–transition-related reason for limited PA was the seasonal influence, with less activity in winter:

During the pandemic, we could not have training sessions, or matches. I did a lot less sport then.

Participant in group 3

My coach created an online group and offered activities: who has time on this day at that time? And we had to see for ourselves if we could make it, and if there were only four of us, it was fun, and if there weren’t even four, it could not take place. Like that, we could continue practice once or twice a week, but sometimes we could not.

Participant in group 4

#### Capabilities, Reasons, Motivators, and Demotivators for PA

With regard to motivators for PA, participants mentioned being together and having fun together as the most important aspect. Participants mentioned making friends and being motivated by others as the drivers of motivation:

You need a bond with your team if you do football or another team sport. If you have bad team spirit... We had a period where our team was no fun to be in...

Participant in group 3

If you join a sport club, it gives you a lot of social contacts as well

Participant in group 2

Other important motivators for PA were the positive mental health effects associated with being active as well as positive physical health effects and adoption of healthy habits. Psychosocial aspects such as achieving mastery and competence or having a purpose in life were also mentioned. Competing with others was not considered an important motivational driver of PA:

You know, when I’m stressed, to clear my head

Participant in group 2

When I’m doing homework and I am getting tired, I take a walk or move around for a bit; they say it helps you concentrate

Participant in group 3

When I’m in a bad mood and I do sport, I am happy again and then my parents are happy as well [laughs]

Participant in group 4

I just like the adrenaline

Participant in group 1

Many participants mentioned psychosocial skills such as confidence, perseverance, self-efficacy, and successful experiences as drivers of PA:

I like it when I can do something and my brother wants to do it as well, it gives me confidence

Participant in group 5

When we are 7-0 behind in a match, it is hard to keep faith; sometimes kids leave the field crying and that does you no good

Participant in group 5

But if I believe in myself, I can perform a lot better

Participant in group 4

Participants also mentioned demotivators to engage in PA. Again, social aspects were the most prominent: unlikeable coaches, teammates, and audiences as important demotivators for (organized) PA. Being judged on performance and a lack of confidence were also mentioned as demotivators:

If you have a bad coach, you feel like going less and if that goes on, you just quit

Participant in group 1

I’m in a mixed football team with boys and girls, but lately all the girls have been ill and then I won’t go either, that influences me. The boys aren’t always nice, are they

Participant in group 5

### Theme 2: Activity and the Social Network

Participants mentioned a range of ways to interact with their social networks regarding PA. First, there were some types of information they would share with others or want to know from others and some information they would not share or want to know. Second, the information shared with, or received from, others had different perceived effects on the participants. In the eyes of the participants, others could not only motivate but also demotivate. BCTs involved were feedback on behavior and performance; social comparison; and providing a descriptive norm, practical support, emotional support, and inspiration.

#### Sharing Information and Hearing From Others

Participants said they wanted to share their information with people they already knew: friends, parents, and siblings. Classmates and peers who they did not know but were on similar performance levels in terms of PA and sport were hardly ever mentioned. No mention was made about wanting to share information with people more distant than direct contacts or people who are not peers:

Some of my classmates are in my team, I share a lot with them about sport, but not with other classmates

Participant from group 5

I talk to my mother about it, and she would ask me about what happened and what I did, so I share with her what I do

Participant in group 2

When talking about what information participants would want to share, they mostly mentioned wanting to share the kind of activity, its intensity, and the amount of time or duration of the activity; however, some participants also mentioned not wanting to share this information. Sharing performance levels, successes, and failures was less popular. Some participants would want to share successes, and few participants would share failures, if they felt the failures were socially acceptable and not too detrimental to their self-image. More participants would not share failures:

I share [information about how often I do sport], but I can imagine that people who move less would enjoy sharing that less

Participant from group 1

But, you know, I would sooner tell someone that I won, or I succeeded, or I am happy or proud about this

Participant from group 4

I would not share how often I do sport, or what I eat, or that kind of thing

Participant from group 2

With respect to wanting to hear from others, similar patterns arose: wanting to know the activity itself, the amount of PA, and the intensity of others’ PA were mentioned often, but participants did not want to hear about others’ failures and overall were not very interested in others’ successes or performance levels:

How much sport people do and what kind. We used to do that, when we were stuck in quarantine, we would ask each other what we were doing now. Just because we could not do any other sport

Participant in group 1

For instance, when they do something successful, that’s nice, but not when they did not succeed

Participant in group 5

Why people sit on the sofa all day and not just enjoy doing a sport, or why some others exercise so much, if there’s a reason for that or if they just enjoy it

Participant in group 2

#### The Effect of the Social Network on PA

Participants felt that people in their social network can act both as drivers and barriers to motivation for PA, depending on the sign of the feedback; positive feedback was seen to be a strong motivator, especially from coaches and friends. However, the detrimental effects of negative feedback were mentioned even more often, with negative feedback from coaches and spectators at sports matches seen as the most harmful. Negative peer judgment and negative gender norms in sports activities played strong roles in negative social effects on PA. Overall, coaches, teammates, parents and siblings, and friends played the biggest role, and teachers, web-based role models, and spectators at sports matches played a lesser role:

That happens with friends too, if you’re doing a sport that they find weird and they give their opinion, it can make you quit

Participant in group 2

And when I tell [them] I enjoy kickboxing, I get reactions like “why do you like that, you are a girl?” and I just don’t care at all about that

Participant in group 3

If the coach says you’re doing great, you tend to believe that because they know what they are talking about. And that makes me try even harder

Participant in group 1

Participants felt that the social network could support PA by providing practical support, almost always in the form of inviting the participant to take part in activities or pushing the participant toward being active. Providing emotional support was seen as another strong facilitator of PA. Furthermore, people from the social network could provide a descriptive norm (showing what activities are “normal”) and provide inspiration (showing what activities are possible). Finally, participants thought that social comparison could be both a driver and a barrier for PA:

I guess I started football because of [other participant]; she said we are looking for more cool girls on the team, and yes, that convinced me to join

Participant in group 2

When people are yelling “come on, you can do it, keep it up!” that helps. It gives you a bit of support. “This time you might not hit the ball right, but next time you’ll do fine.”

Participant in group 1

### Theme 3: Activity and Digital Technology

Participants mentioned a broad range of social media platforms that they regularly used. Snapchat (n=8), WhatsApp (n=7), Instagram (n=7), TikTok (n=7), and YouTube (n=5) were often mentioned. Participants were aware of apps (6 mentions) and gadgets (7 mentions) for PA, and most participants had used at least one of those at some point to track PA or perform workouts. Participants also mentioned YouTube (6 times) as a provider of inspirational videos. Social media, as well as gaming and Netflix, were recognized as distractors (16 mentions) and stimulators (21 mentions) of PA:

If you follow more people on YouTube who post about sport and stuff, it makes you feel like doing it yourself more. You can push yourself to do it like that.

Participant in group 3

I notice I go outside to do sport less because I get side-tracked by other stuff, like when I just did my homework and I’m like now is the time to watch some TV or play a game, and when I’m sitting on the sofa, I find it really hard to get up again and go outside and get active.

Participant in group 4

When considering what BCTs would encourage them to engage with digital technology for PA, the participants mentioned goal setting, social and interactive techniques, notifications and reminders, feedback on behavior, and rewards for use and goal attainment. Competition was generally considered uninteresting; if any competition at all was taking place, participants found it important that they competed with peers of a similar level.

#### Goal Setting and Action Planning

Most participants saw goal setting and support in action planning as effective BCTs to encourage PA. Setting your own goals, tailored to one’s own needs and attainment level, and personalized action plans were seen as important for uptake and sustained engagement. Participants especially found setting their own challenges and being able to choose from different activities to be important. Keeping steps small and feasible, support in keeping up streaks and other ways to form habits, and being able to adjust goals also emerged:

I would not use preset groups, but if I can create my own group and set my own goals, I would like to do this much next month or next week, like that.

Participant in group 1

Or the app could tell you “you should do this and that, and this is for the leg muscles, and this is for the stomach, and if you do this so often, you will go this direction” or something like that.

Participant in group 3

I guess it is important to set your own goals, or else if it says you need to do 5000 steps, you will keep on receiving notifications that you still need to walk, and then you will want to remove it.

Participant in group 4

#### Social and Interactive Aspects

The second group of BCTs mentioned often by the participants was social and interactive techniques. Autonomy, that is, creating one’s own groups; inviting friends; and being able to give each other challenges; and fairness, that is, competition only against peers of similar level, were seen as important:

Adding your own groups, and then play who gets the most points. You can do that with your own friend group—“let’s use this app and we’ll do this much this month.” Who gets most points wins?

Participant from group 1

Last year, my group eight (last year of primary school) used the [Popular Dutch app for suggesting walks], and everybody started taking walks and comparing who went furthest.

Participant in group 5

Like, I would challenge [other participant], saying here, I managed to do this, will you beat that? That sort of thing. You keep encouraging each other like that.

Participant from group 2

#### Other BCTs

Participants mentioned rewards for the use of the app and for goal attainment as potential drivers of engagement: the ability to unlock premium content; receive points, badges, and web-based stickers; or receive products and gadgets as a reward were mentioned as different reward possibilities, with monetary incentives seen as less motivating. Feedback on performance, especially personalized feedback, and the use of notifications and reminders to keep up engagement were also seen as potential drivers of engagement:

It could give you an overview of where you have been last month. “You have been using this app for a month now, would you want to see where you ran?” Red and dark yellow can be where you went a lot, and light yellow where you went less.

Participant in group 4

It can give you points, and you can use them to buy an avatar that runs your route with you, like the car in Google Maps.

Participant in group 1

We used that [popular Dutch app for suggesting walks] and you kept on getting, what was it again, some kind of medal or something—“you achieved this, you will get this next time.” You do want to collect them all, you know?

Participant in group 2

#### Downsides to Technology Use

Participants also saw downsides to digital solutions for PA. The potential dangers of addiction and overuse were seen as important downsides, as were having to deal with negative comments and responses, privacy issues, potential discord and friction, and demotivation when missing goals:

I know that some people can get very insecure when they see stuff on social media, like some people can do great things and they cannot

Participant in group 5

And you should not get 800 messages, because it’ll make you remove the app very quickly

Participant in group 2

For example, when you do a lot of steps and end up higher on the leader board, that works, but if you don’t get higher or don’t get on the leader board and it feels like you lose, it will make you stop playing at once

Participant in group 4

I would not make it super-competitive; it has to remain fun, and you’ll end up doing only this thing

Participant from group 5

#### User Experience Aspects

Finally, the participants mentioned the importance of a good user experience and the design of PA technology. Ease of use, attractive design, and not having in-app commercials all play a role in uptake and sustained use:

Well, I think it’s a definite plus if the app isn’t too complicated. Because when you install an app where everything is difficult at first, you’ll soon think “never mind, I’m not doing all this”

Participant in group 2

Yes, when you’re doing great, and then there’s an ad for sweets or chocolate...

Participant in group 3

### Saturation

The analysis of the base set of 3 group sessions revealed 112 codes in 3 main themes (*Physical activity*, *The social network in physical activity*, and *Digital technology for physical activity*) and 26 subthemes. Including the next run of 2 group sessions added only 3 new codes (2.97%), which was below the predefined cutoff point of 5%; no new themes and subthemes occurred. This means that thematic and code saturation can be assumed to have been achieved.

## Discussion

### Principal Findings

This study aimed to evaluate what adolescents who had just made the transition from primary to secondary school think and feel about facilitators and barriers to active living, the role of their social network in active living, and the potential for digital technology. The study showed that all participants were physically active to a certain extent, with most engaging in regular sports activities in addition to their obligatory school physical education class. Participants felt that the transition to secondary school made it harder to be physically active due to time constraints, such as more time needed to do schoolwork. They also mentioned the pandemic as a limiting factor, although at the same time, they mentioned that regular activities were taken up as soon as lockdowns and other measures were abandoned. Psychosocial skills such as confidence were regarded as the most important capabilities for PA, and benefits for mental and physical health were mentioned as motivators. Importantly, participants regarded social benefits as strong motivators for PA: being together, making friends, and having fun together. Apart from the motivational effect, social networks were also mentioned as demotivators. Specifically, unlikeable coaches and teammates were the most important demotivating forces. These results are in line with recent literature [83,84], which cites motivation; perceptions of competence and body image; fun; influence of friends, family, and physical education teachers; and environmental PA opportunities as the strongest drivers of active living. An important driver from the literature that was not mentioned by any of the participants was their attitude toward PA; a positive attitude seemed to be given in this sample and was not explicitly mentioned.

Regarding the influence of social networks on PA, the participants viewed sharing information with others as important. Participants were willing to share positive experiences, mostly with close networks (friends, classmates, and parents), but would not share negative experiences or personal information. Some participants would share information such as sports frequency, while others would not. With respect to receiving information from others, participants would mostly want to know what others do as inspiration for their own activities, that is, what others do as a descriptive norm for PA or to know what activities they can do together (social motivation). They were not interested in the negative experiences of others. These insights have no direct counterpart in the current literature. Few studies on sharing PA experiences among adolescents exist, and those studies have a smaller sample size and do not justify their conclusions because they concern the usability of a specific digital application [85]. However, these studies show similar patterns: small groups, close friends, and positive experiences. In line with our findings on negative feedback, a recent study [43] showed that receiving social support tokens (“Kudos”) can augment PA in adults, but receiving kudos from individuals with a low running intensity negatively impacted others’ activity. Thus, our results, in combination with those of recent literature, clearly demonstrate that the effect of the social network is not only beneficial but can also have a negative impact on behavior. These combined findings warrant further study to refine insights into the role of social networks in relation to the specific preferences of adolescents.

Participants mentioned a range of BCTs through which their social networks helped them in active living. Emotional support is important, and it is mostly the coach and friends or teammates who provide it, as well as the family members. Coaches, teammates, and physical education teachers can also have demotivating influences. Practical support can come from a wider social network; this includes triggers to “get you off the sofa” or facilitate locations and activities. Thus, feedback from the social network is a two-sided sword. Participants stated that negative feedback and judgment, which often relate to gender norms, can be detrimental to motivation, whereas positive feedback boosts confidence and motivation. Social comparison also plays a role; participants wanted to compare themselves to people who had similar interests and capabilities and disliked “perfect” images on Instagram. The role of the social network in active living described by the participants of this study is similar to that reported in recent literature, where inspiration, the impact of positive feedback [86], positive social support and the role of gender norms [21], and the role of emotional support [87] are well-documented drivers of PA.

With respect to ICT-based solutions to promote active living, the participants mentioned many well-known BCTs from the literature. The most prominent were personalized feedback, personalization of goals and challenges, and notification that strikes a careful balance between providing positive stimulation and avoiding becoming bothersome, and rewards. If competition was included, participants felt that it should be with friends, their own groups, or people at a similar level. Competition was mostly mentioned in a social context, with fun being more important than the competition itself. Finally, attractiveness, ease of use, and personalization were mentioned as important design features. These insights into which BCT participants believed to affect their engagement in PA reflect recent overviews from the literature on what works for engagement [72]. The participants were apprehensive about negative social interactions in a web-based environment. In addition, they recognized that apps are quite easy to quit, can lead to addictive behavior, and can be demotivating in case of distracting contents or insurmountable goals. Some privacy issues, such as location, were also discussed. This is in line with research [88] that shows that adolescents are well aware of the risks of cyberbullying, addiction, and problems with their self-image.

### Limitations

Although the results of this study provide an overview of what participants thought and felt about social networks, digital media, and PA, they are by no means exhaustive, given the study design that had focus group discussions rather than questionnaires or other quantitative approaches. An example is the mention of social media use: WhatsApp received 7 mentions, whereas it is likely that all participants used this platform. Participants often added to others’ answers, that is, if one participant already mentioned something (such as using a social media platform), others did not feel the need to mention it as well. Therefore, this study is not an overview of which social media and how frequently adolescents use it, but it does provide information on which channels are useful when working with adolescents. Another limitation is the setting of the study: the experiences that the participants mentioned may be typical only for Dutch adolescents aged 12 to 14 years and may lack validity in other societal settings. However, we expect the themes mentioned by the participants in this study to hold true for children and adolescents across many populations. Further research in other settings can test this expectation.

Every scientific study is subject to bias, and this study is no exception. A likely bias is attributed to the self-selection of participants; only those who expressed interest were invited to participate. This may have led to a skewed distribution of PA in the sample. In fact, all participants were physically active, which may have been unrealistic. This potential skewness may also explain why attitudes toward PA were not a topic of discussion. However, as PA also included obligatory physical education, it is not unreasonable to assume that the participants’ PA reflects that of the general adolescent population. Recent figures show that 75.8% of adolescents aged 12 to 18 years in the Netherlands participate in sports at least weekly [89], and these results do not take physical education participation into account. However, further research should focus on the stratification of different levels of PA. Cultural influences might have been at play in some of the results, for instance, in how the participants rated competition. Their relative disinterest in competition may be partly influenced by their cultural background, and adolescents from other cultures might be more inclined to like competing with their peers [90]. In addition, these results have implications for individuals who do not exercise. For them, the positive effects of the social network shown in our study may also be implemented in a behavioral change intervention that targets the group of “nonmovers.” However, including the views of adolescents who do not participate in sports is needed to validate this implication.

Literature shows that there is a need for more specific research into the effect of socioeconomic status and sex differences on active living in adolescents [83]. However, this study did not provide such stratified results because first, we had no access to data to establish socioeconomic status group membership for our participants; second, the sample size did not allow stratifications to be made possible without making concessions to power and validity. Further research could replicate this study using priority sample groups. For example, Spruijtenburg et al [17] showed in a large sample of first-year secondary school students (n=386) that motives, perceived competence, encouragement, and motor skills were significantly associated with participation in organized sports. This kind of encouragement may also be delivered in a digital form, for example, by providing kudos as in the Franken et al [43] study.

### Conclusions

This paper shows that adolescents felt that their social network was and can be a strong driver of PA and active living. They were willing to use ICT-based solutions that make use of social networks for PA, as long as they involve their peers, close friends, and teammates. They felt it was fine to share some information such as their successes, and sometimes their general activity. They would not share their failures and would not be interested in competition unless it was mostly about having fun together while competing with peers of similar performance levels. ICT-based solutions should have personalized goals and feedback, provide the ability to create their own groups and set their own challenges, and facilitate having fun and meeting each other.

The results of this study could be of interest to anyone (including scientists, teachers, clinicians (physiotherapists and occupational therapists), and social workers) currently undertaking or planning interventions to support PA in adolescents. The results show that there are opportunities to develop ICT-based, social network–including interventions for active living. These should be open solutions that help adolescents in goal setting, reaching out to friends, setting each other challenges, being together, and enjoying themselves together, all through the lens of PA. The results show not only that it may be fruitful to move away from the individual approach, which is still too common in digital technology for PA, but also that these technologies should aim for more cooperation than competition. Such ICT-based solutions could support adolescents in having a more active lifestyle and overcome the potential negative effects of the transition to secondary school, setting the stage for sustained activity in later life.

## Appendix

Multimedia Appendix 1 Interview guide.

## Abbreviations

BCT: behavior change technique
ICT: information and communication technology
PA: physical activity

## Data Availability

The datasets generated and analyzed during this study are not publicly available due to privacy considerations but are available from the corresponding author on reasonable request.
