# Supplementary material for: The Effect of Social Networks on Active Living in Adolescents: Qualitative Focus Group Study
Source: JMIR Form Res. 2023 Oct 5;7:e46350. doi: 10.2196/46350 (PMC10587806; doi:10.2196/46350)
Supplement: Multimedia Appendix 1 [file formative_v7i1e46350_app1.docx]

**Appendix 1: Interview guide**

**Introduction**
Moderator introduces researchers, their tasks, and the goal of the research
Moderator introduces main interview topics:

1) Sport and physical activity

2) Online social network and physical activity

3) Technology use and physical activity

4) Social media and physical activity

Moderator underlines that interviews will be processed anonymously, explains how; explains recording, once more asking for permission to record the session.

**1) Sport and physical activity**

**Starter question**

When we look at sport and physical activity, what do you think of? What comes to mind?

**Potential follow-up questions**

- Why do you do sport, why not?
- What sport(s) do you do?
- How often?
- Did you change the amount of sport in the past year?
- What reasons did you have to quit, what reasons to keep on doing sport?
- What could help you be more active?

**2 (Online) social network**

**Starter question**

When we look at your (online) social network and physical activity, what do you think of? What comes to mind?

**Potential follow-up questions**

Which persons are important for your physical activity?

How do they influence your activity?

Do you have examples of persons who affect your physical activity negatively? How?

Do you have examples of persons who affect your physical activity positively? How?

Does physical activity play a role in feeling part of a group? How does that work for you?

- What kind of information would you like to know of your peers when it comes to physical activity? And what not?

- What can your social network do or mean for you in order to increase your physical activity?

**3) Technology use and physical activity**

**Starter question**

When we look at digital and information technology and physical activity, what do you think of? What comes to mind?

**Potential follow-up questions**

Have you ever used an app for sport or physical activity? Examples?

If we were to make an app to help you be more active, what should the app definitely contain?

What would help you be more active, and what not?

Do you think such an app would work for you? Why or why not?

**4) Social media and physical activity**

When we look at social media and physical activity, what do you think of? What comes to mind?

**Potential follow-up questions**

What social media do you use?

What impact does social media have on your physical activity? What benefits, what downsides?

How can social media motivate you to be more active?

Can you give examples of moments where social media affected your physical activity positively? And negatively?

What would you like to see in social media about physical activity?

**Closing off and thank you**

*Summarise conversation, ask if people have any other remarks, closing off and thank you*
